# Vaccination Denounced

**Published:** 1895-11

**Authors:** T. Dwight Stow


### VACCINATION DENOUNCED.

Resolutions adopted by the Central New York Homœopathic Medical Society, in session in the city of Rochester, N. Y., June 20th, 1895:

WHEREAS, It is a cardinal principle of our faith, that, except for the purpose of proving drugs upon the healthy, it is bad practice to induce morbid or morbific conditions in the human organism, when in health; also, that it is unphilosophical and contrary to physiological law to prescribe any remedy for the cure of the sick save a homœopathic remedy; and

WHEREAS, We believe it to be not only absurd and injurious, but little short of criminal, to inoculate or to vaccinate human beings, or even brutes, with the morbific products of any disease, on the mistaken and unwarrantable hypothesis of protection against variola, diphtheria, tuberculosis, *et id omne genus*, taking reason and experience as our guide; therefore,

*Resolved*, That we hail the increasing evidence of opposition to vaccination with profound satisfaction, and welcome the advent of the new national Anti-Vaccination Association, organized at the Fifth Avenue Hotel, New York, on the 5th day of June, 1895; also Dr. Foote's *Journal of Health*, the *Anti-Vaccination News*, of New York, and the *Journal of Hygeo-Therapy* as marked features of and powerful factors in the just war against all superstition, barbaric fetichism, or therapeutic conjecture.

*Resolved,* That compulsory vaccination, or State compulsion in matters pertaining to religion or medicine, or of freedom of speech or of the press, is unjustifiable in morals and in law, and we will do all in our power, by worthy and just endeavor, to oppose them, and we will aid to the extent of our ability all efforts made by others in the same direction.

*Resolved,* That a copy of these resolutions be furnished the *Medical Advance;* HOMŒOPATHIC PHYSICIAN; *Anti-Vaccination News,* of New York; Dr. Foote's *Journal of Health;* the *Journal of Hygeo-Therapy,* of Kokomo, Ind.

T. DWIGHT STOW.

